# Rate of nicotine metabolism on risk of myocardial infarction among people with HIV who smoke cigarettes

**DOI:** 10.1371/journal.pone.0356296

**Published:** 2026-08-18

**Authors:** Derartu Ahmed, Warren B. Bilker, Xiaoyan Han, Rachel F. Tyndale, Jessica Merlin, Stephen E. Kimmel, Jeffrey Martin, Michael Plankey, Katerina Christopoulos, Leila S. Hojat, Laura Bamford, Sonia Napravnik, Robert Schnoll, Rebecca Ashare, Robert Gross

**Affiliations:** 1 University of Pennsylvania Perelman School of Medicine, Department of Biostatistics, Epidemiology and Informatics, Philadelphia, Pennsylvania, United States of America; 2 University of Toronto, Department of Pharmacology and Toxicology, Ontario, Canada; 3 Campbell Family Mental Health Research Institute, Centre for Addiction and Mental Health, Ontario, Canada; 4 University of Toronto, Department of Psychiatry, Ontario, Canada; 5 University of Pittsburgh, Pittsburgh, Pennsylvania, United States of America; 6 University of Florida, Department of Epidemiology, GainesvilleFlorida, United States of America; 7 University of California San Francisco, San Francisco, California, United States of America; 8 Georgetown Medical Center, Department of Medicine, Washington, District of Columbia, United States of America; 9 Case Western Reserve University School of Medicine, Cleveland, Ohio, United States of America; 10 University of California San Diego School of Medicine, San Diego, California, United States of America; 11 University of North Carolina at Chapel Hill, Department of Medicine, Chapel Hill, North Carolina, United States of America; 12 University of Pennsylvania Perelman School of Medicine, Department of Psychiatry, Philadelphia, Pennsylvania, United States of America; 13 The State University of New York, Department of Psychology, Buffalo, New York, United States of America; Universidade Federal do Para, BRAZIL

## Abstract

**Background:**

The prevalence of cigarette use in people with HIV (PWH) is 2–3 times higher than the general population. Cigarette use increases risk of myocardial infarction (MI). Faster nicotine metabolism, quantified by the nicotine metabolite ratio (NMR) is associated with greater risk for nicotine dependence and lung cancer, but its association with MI is unknown.

**Methods:**

We conducted a nested case-control study within the Center for AIDS Research Network of Integrated Clinical Systems (CNICS) cohort. Cases were PWH who reported cigarette use with incident adjudicated MI between 2003 and 2020 and available plasma samples; cigarette-smoking controls were selected by incidence density sampling, matched on age, race, birth sex, and plasma HIV RNA level. Conditional logistic regression was used to estimate odds ratios (OR) for the association of NMR and MI.

**Results:**

We identified 135 cases with MI and 252 controls. Median (IQR) NMR was greater in cases [0.51 (0.36, 0.73)] than in controls [0.47 (0.30, 0.70)]. In conditional logistic regression, the odds of having a high NMR were 1.4 times greater among MI cases than controls, but not at a statistically significant level (OR: 1.4, 95% CI: 0.97–1.9). This estimate did not substantively change after further adjustment for statin use, hypertension, and diabetes (OR: 1.4, 95% CI: 0.92–2.0).

**Conclusions:**

There is a small-magnitude association between NMR and MI, which was not statistically significant. NMR remains a potential biomarker for MI risk among PWH. Further investigation is needed to estimate the precise effect of NMR on MI in PWH.

## Introduction

Cigarette use [1] and HIV [2] are well-established risk factors for myocardial infarction (MI). Among PWH, the prevalence of cigarette use is approximately twice that of the general population [3], and cigarette use doubles the risk of cardiovascular complications in PWH [4]. The reasons for increased cigarette use are not fully understood, but may relate to nicotine metabolism [5].

Nicotine dependence is related to the rate of nicotine metabolism [6]. Nicotine is primarily metabolized by CYP2A6 to cotinine (80%) and then to 3-hydroxycotinine (3-HC) [7]. Enzymatic activity of CYP2A6 is measured using the nicotine metabolite ratio (NMR), calculated as the cotinine concentration divided by 3-HC concentration [8]. The NMR reflects both genetic and environmental influences on CYP2A6 and thus nicotine clearance [8]. Measurement of NMR using plasma is reliable and valid, and stable over time [9–11].

A higher NMR indicates faster nicotine metabolism, which is associated with higher cigarette use, lower cessation rates, greater total puff volume and higher levels of carcinogens [12–14] Additionally, among PWH who use cigarettes, NMR increases after viral suppression, with doubling among those on efavirenz-based regimens [5,15] The faster nicotine clearance may therefore increase exposure to toxins in cigarette that exacerbate cardiovascular risk. If an association between faster nicotine metabolism and higher MI rates in PWH were identified, NMR could become a novel biomarker of cardiovascular risk and would further underscore the importance of smoking cessation in this population. We aimed to determine whether NMR is associated with MI in PWH.

## Materials and methods

### Study design

We conducted a matched nested case-control study to assess the association between NMR and MI in people who use cigarettes with HIV. We used data from the Center for AIDS Research (CFAR) Network of Integrated Clinical Systems (CNICS) cohort. Initiated in January 1995, CNICS is a prospective clinical cohort of > 49,000 PWH receiving care at ten CFAR sites nationally [16]. Seven of the CNICS sites were included in these analyses: Case Western Reserve University, Johns Hopkins University, University of Alabama at Birmingham, University of California, San Diego, University of California, San Francisco, University of North Carolina at Chapel Hill, and University of Washington. CNICS captures clinical data and self-reported measures. Plasma samples in the CNICS have been stored at −80 C since its inception.

Participants were selected from individuals with self-reported regular cigarette use, defined as smoking on at least two consecutive time points, a minimum of one year apart, as well as at all assessed intervening time points. Additionally, they had to have aviremic plasma sample available for NMR testing. Cases included all individuals who had an MI and had a plasma sample when they reported cigarette use. Controls were selected using incidence density sampling. Controls were matched to the cases based on site, age, race, sex, viral load status (at time of MI), and calendar time (within the same 365-day period) when cases had MI. MI has been adjudicated in the CNICS cohort [17]. Individuals diagnosed with MI, which could not be confirmed or with a reported cardiovascular disease equivalent, which had not been validated in CNICS, were excluded as cases or controls.

### Outcome and covariate measurement and definitions

Standard liquid chromatography-tandem mass spectrometry (LC-MS) was used to measure both cotinine and 3-HC [10]. A cotinine value > 10 ng/mL confirmed cigarette use [18]. The NMR was treated as a continuous measure, and the log of NMR was reported because the NMR was not normally distributed. We examined demographic characteristics, including age, race, ethnicity, and birth sex, clinical characteristics including CD4 count, history of hypertension, diabetes, and statin medication use. Given the association between the duration and level of exposure to HIV viremia with adverse outcomes, we estimated the HIV viremia copy years for each participant at the time of MI or censored using the trapezoid method applied to all available viral loads (See Supplementary Information for the trapezoid method) [19]. To account for the effect of efavirenz’s on the NMR, we calculated the NMR as a weighted average which was computed by summing the NMR on efavirenz x time on efavirenz + NMR off efavirenz x time off efavirenz divided by total cigarette use duration, which we assumed to be the individual’s current age minus 16 [5,15]. Cigarette use was imputed for all participants to have begun at age 16 [20]. We considered the pre-ART NMR to be the same as the non-efavirenz containing regimen NMR given the relatively small increases in NMR in individuals initiating non-efavirenz containing regimens [5].

### Statistical analysis and sample size

Baseline characteristics were compared using chi-squared tests for categorical variables and t tests or rank sum tests for continuous variables. Normality was assessed using the Shapiro-Wilk and histograms. Conditional logistic regression was used to compare NMR between those with and without MI. Potential confounders were selected based on biological plausibility. We targeted a sample size of 145 cases with a goal of 2:1 control to cases for 80% power to detect a 0.1 value difference in NMR between cases and controls with a p-value of 0.05. Sensitivity analysis was conducted to assess the impact of excluding participants with missingness and test the assumptions used in the weighted NMR calculation.

### Ethics statement

The study was reviewed and approved by the University of Pennsylvania and University of Toronto institutional review board. The required for written informed consent was waived by the Ethics Committee. All data and specimens were anonymized by CNICS and therefore, the authors had no access to identifiable personal. Data access for research purposes was obtained on August 01, 2019. Additional data were obtained afterward to ensure that the enrollment included individuals who had MIs in 2020.

## Results

### Characteristics of cases and controls

We identified 135 participants (mean age 51; 76% male) with myocardial infarction (MI) occurring between 2003 and 2020. We identified 252 matched controls (mean age 50 years; 76% male) and were unable to find a suitable second matched control for 18 cases. Two controls samples were excluded due to missing NMR. There was little missingness in the data with diastolic and systolic pressure having <2% and total cholesterol with 22% missingness in cases and 23% in controls. Table 1 summarizes participants’ baseline demographic characteristics. Cases and controls did not differ significantly at baseline, except for cases having higher rates of hypertension, diabetes, and statin medication use, compared with controls.

**Table 1:** Participant demographic characteristics.

Demographics	Cases (N = 135)	Control (N = 252)	P-Values
Age (Years)	51.2 (8.1)	50.2 (9.4)	0.065
Gender male, n (%)	103 (76%)	192 (76%)	
Race			0.986
White	52 (39%)	99 (39%)	
Black	82 (61%)	152 (60%)	
Multiracial	1 (0.7%)	0 (0%)	
Unknown	0 (0%)	1 (0.4%)	
Ever had Hypertension			**<.001**
No	50 (37%)	177 (70%)	
Yes	85 (63%)	75 (30%)	
Diabetes			**0.009**
No	106 (79%)	222 (88%)	
Yes	29 (22%)	29 (12%)	
Missing	0 (0%)	1(0.4%)	
Ever used medication statin			**<.001**
No	89 (66%)	208 (83%)	
Yes	46 (34%)	44 (18%)	
HIV RNA (copies/ml)			0.809
Undetectable or < 400	103 (76%)	194 (77%)	
400–999	4 (3.0%)	7 (3%)	
1000 to 9,999	7 (5%)	12 (5%)	
>10,000	21 (17%)	39 (15%)	
CD4 Counts (cells/cm^3^), IQR	427.0 (228.0, 399.8)	506.5 (288, 718)	0.609
Systolic Pressure (mm Hg) (sd)	129.8 (21.7)	126.8 (17.8)	0.173
Diastolic Pressure (mm Hg) (sd)	80.5 (13.1)	78.8 (11.2)	0.197
Total Cholesterol (IQR)^**+**^	170.0 (146,196)	167.0 (141, 203)	0.705
HIV Acquisition Group			0.747
MSM & not IDU	55 (41%)	99 (39%)	
IDU & not MSM	31 (23%)	56 (22%)	
IDU & MSM	12 (9%)	16 (6%)	
Heterosexual contact	37 (27%)	73 (29%)	
Other risk	0 (0%)	8 (3%)	

MSM = Men Who have Sex with Men

IDU = Injection Drug Use

*IQR, interquartile range reported if data not normally distributed

*The Fisher exact test for count data.

* Wilcoxon rank sum test for continuous data not normally distributed

* Independent t-test for continuous data normally distributed continuous

* Matched on age, site, race, sex, and viral load status at the time of MI

+ 30 out of 135 cases and 57 out of 252 controls were missing total cholesterol

*blood pressure, viral load, and CD4 count reflect the last recorded value prior to the MI event or censoring.

*Viral load measurements were conducted at the time of the NMR sampling.

### NMR and Myocardial infarction

Median (IQR) NMR was greater in those with MI [0.51 (0.36 0.73)] than controls [0.47 (0.30, 0.70)]. In the conditional logistic regression model, patients with an MI had higher NMR than controls, but the difference was not statistically significant (Table 2). Patients with an MI also had a greater history of statin use, hypertension, and diabetes than controls. Adjusting for these factors did not substantively change the NMR-MI relationship (OR: 1.4, 95% CI: 0.92–2.00).

**Table 2:** Conditional logistic regression analysis for factors associated with MI.

Variables	Univariate Analysis		Multivariable Analysis	
	Estimate OR (95% CI)	P-value	Estimate OR (95% CI)	P-value
Ever used medication statin		0.0005		0.137
No	Ref		Ref	
Yes	2.47(1.49-4.09)		1.53 (0.873- 2.677)	
Ever had Hypertension		<.0001		<.001
No	Ref		Ref	
Yes	4.10 (2.53-6.63)		3.38 (2.04- 5.61)	
Diabetes		0.0085		0.119
No	Ref		Ref	
Yes	2.17 (1.22-3.86)		1.66 (0.88-3.14)	
Log of NMR	1.37 (0.97-1.94)	0.0753	1.35 (0.92-1.99)	0.122

OR= Odds Ratio, Ref. = Reference category.

Univariate analyses were matched on study site, age, race, sex, viral load status at the time of myocardial infarction, and calendar time.

Multivariate analyses the variables we matched on (on study site, age, race, sex, viral load status at the time of myocardial infarction, and calendar time) as well as adjustment for statin medication use, history of hypertension, and diabetes status.

*see methods for calculation of HIV viremia copy-year.

Given the strong effect of efavirenz on changes in NMR, we calculated the NMR as a weighted average which was computed by summing the NMR on efavirenz x time on efavirenz + NMR off efavirenz x time off efavirenz divided by total smoking duration.

The relationship between MI and NMR did not exhibit a simple and consistent pattern. Therefore, to further investigate this, we categorized NMR into four quartiles, with each group containing the same number of people and ranked from lowest to highest. The odds of having NMR in the third versus first quartile were 1.79 times higher among cases than controls (S1 Table), although not a statistically significant effect (OR: 1.79, 95% CI: 0.92–3.5).

### Sensitivity analysis

To assess the robustness of our main findings, we conducted sensitivity analyses using an alternative definition of NMR (S3 Table). The effect size of NMR risk on MI remained similar (OR: 1.3, 95 CI: 0.9–1.9). Excluding participants missing total cholesterol did not change the study conclusions (S2 Table).

## Discussion

We examined the relationship between MI and NMR among PWH who use cigarette. Although the association between MI and NMR was not statistically significant, the elevated risk and wide confidence interval indicate substantial variability in the data. Therefore, the findings remain inconclusive. Our findings may reflect the complex interplay between MI, HIV, ART, and cigarette use, as well as some of the characteristics of our study design.

A systematic review and meta-analysis found that HIV infection, low CD4, high plasma viral load, and cumulative ART use in general were associated with increased risk of MI. Cigarette use was notably not considered due to inconsistent reporting in included studies [21]. Similarly, others have reported on the association between the duration and level of exposure to HIV viremia with adverse outcomes [19]. To account for this, we estimated the HIV viremia copy years for each participant at the time of MI or censor for all available viral loads. However, we lacked data on viral load from the time of infection to the study enrollment, as the duration of HIV prior to enrollment in the CNICS database was unknown. Consequently, we could not account for differences arising from variations in the length of HIV infections. Nevertheless, given that the majority of our population has suppressed viral load, indicating adherence to ART, it is likely that they were at least somewhat protected against MI.

Another challenge is ART, medications are known to increase NMR, in particular efavirenz [5,15]. This suggests that the choice of ART may impact nicotine metabolism. To adjust for differences in NMR that might be due to ART regimen, we calculated the NMR as a weighted average. Since cigarette use duration is estimated, this measure of NMR is potentially imperfect, as it does not fully account for the actual time spent on each ART regimen.

Our study has several potential limitations. We did not have NMR measurements before participants started ART. Our assumptions of the weighted average of NMR prior to and during ART may have introduced bias, as it assumed smoking at age 16 and may not capture NMR differences due to ART variations. Reassuringly, our sensitivity analysis to explore the impact of these assumptions did not affect the overall finding. Despite our sensitivity analysis, the lack of a clear dose-response and wide confidence intervals from categorized NMR results suggests these findings may reflect limited power, potentially due to strict matching leading to exclusion of two controls, difficulty disentangling MI, HIV, ART, cigarette use, or noted study design limitations.

Despite these limitations, the study has several strengths. The MI cases had been adjudicated, and it is unlikely that a control followed in CNICS had an undetected MI [17]. Including all participants who reported cigarette use eliminates confounding by cigarette use status. Matching on key variables reduced confounding, enhancing the isolation of NMR effects. Sensitivity analysis excluding participants missing total cholesterol showed results were not substantially impacted by missing data.

## Conclusions

Our study does not provide strong evidence of a clear association between MI and NMR. However, given the higher NMR value among the cases, future studies are needed to investigate potential biological mechanisms underlying cardiovascular risk in PWH who smoke.

## Supporting information

S1 Table Conditional logistic regression analysis for factors associated with MI using NMR quartiles. (DOCX)

S2 Table Sensitivity analysis for total cholesterol: Impact of missing data on NMR estimate. (DOCX)

S3 Table Conditional logistic regression analysis for factors associated with mi using difference weighted NMR. (DOCX)

S1 File Trapezoidal rule. (DOCX)
