# Supplementary material for: Rate of nicotine metabolism on risk of myocardial infarction among people with HIV who smoke cigarettes
Source: PLoS One. 2026 Aug 18;21(8):e0356296. doi: 10.1371/journal.pone.0356296 (PMC13485018; doi:10.1371/journal.pone.0356296)
Supplement: Supplementary file 1 [file pone.0356296.s001.docx]

| Supplementary Table 1. Conditional Logistic Regression Analysis for factors associated with MI using NMR quartiles | | |
| --- | --- | --- |
| Variables |  | |
|  | Estimate OR (95% CI) | P-value |
| Ever used medication statin |  | 0.117 |
| No | Ref |  |
| Yes | 1.58 (0.89- 2.81) |  |
| Ever had Hypertension |  | <.001 |
| No | Ref |  |
| Yes | 3.19 (1.91-5.34) |  |
| Diabetes |  | 0.234 |
| No | Ref |  |
| Yes | 1.49 (0.77-2.85) |  |
| NMR |  |  |
| Q1 | Ref |  |
| Q2 | 1.23 (0.63-2.38) | 0.549 |
| Q3 | 1.79 (0.924-3.45) | 0.085 |
| Q4 | 1.24 (0.622-2.48) | 0.539 |
| OR= Odds Ratio, Ref. =Reference category, Q=quartile, the division was based on NMR values from our dataset, ranked from lowest to highest and divided into equal groups. | | |
