# Supplementary material for: Rate of nicotine metabolism on risk of myocardial infarction among people with HIV who smoke cigarettes
Source: PLoS One. 2026 Aug 18;21(8):e0356296. doi: 10.1371/journal.pone.0356296 (PMC13485018; doi:10.1371/journal.pone.0356296)
Supplement: Supplementary file 2 [file pone.0356296.s002.docx]

**Sensitivity Analysis for Total Cholesterol: Impact of Missing Data on NMR Estimate**

For total cholesterol, a sensitivity analysis was conducted by comparing the model with and without participants missing this variable, with no change in direction or significance.

| Supplementary Table 2. Sensitivity Analysis for Total Cholesterol: Impact of Missing Data on NMR Estimate | | |
| --- | --- | --- |
| Variables | **Excluding participants with missing Cholesterol values** | **Full Sample** |
|  | Estimate OR (95% CI) | Estimate OR (95% CI) |
| Ever used medication statin |  |  |
| No | Ref | Ref |
| Yes | 1.69 (0.891-3.20) | 1.53 (0.873- 2.677) |
| Ever had Hypertension |  |  |
| No | Ref | Ref |
| Yes | 3.23 (1.77-5.88) | 3.38 (2.04- 5.61) |
| Diabetes |  |  |
| No | Ref | Ref |
| Yes | 2.01 (0.976-4.13) | 1.66 (0.88-3.14) |
| Log of NMR | 1.078 (0.682 -1.70) | 1.35 (0.92-1.99) |
| OR= Odds Ratio, Ref. =Reference category  Variables we matched on study site, age, race, sex, viral load status at the time of myocardial infarction, and calendar time, as well as adjustment for statin medication use, history of hypertension, and diabetes status.  + 30 out of 135 cases and 57 out of 252 controls were missing total cholesterol | | |
