# Supplementary material for: Rate of nicotine metabolism on risk of myocardial infarction among people with HIV who smoke cigarettes
Source: PLoS One. 2026 Aug 18;21(8):e0356296. doi: 10.1371/journal.pone.0356296 (PMC13485018; doi:10.1371/journal.pone.0356296)
Supplement: Supplementary file 3 [file pone.0356296.s003.docx]

| Supplementary Table 3. Conditional Logistic Regression Analysis for factors associated with MI using difference weighted NMR | | |
| --- | --- | --- |
| Variables |  | |
|  | Estimate OR (95% CI) | P-value |
| Ever used medication statin |  | 0.127 |
| No | Ref |  |
| Yes | 1.54 (0.883-2.71) |  |
| Ever had Hypertension |  | <.001 |
| No | Ref |  |
| Yes | 3.34 (2.01-5.55) |  |
| Diabetes |  | 0.123 |
| No | Ref |  |
| Yes | 1.65 (0.87-3.1) |  |
| Log of NMR | 1.322 (0.901-1.94) | 0.154 |
| OR= Odds Ratio, Ref. =Reference category. NMR = efavirenz x time on efavirenz + NMR off efavirenz x time off efavirenz divided by follow up duration to adjust for differences due to ART drug use. | | |
