# Supplementary material for: Rate of nicotine metabolism on risk of myocardial infarction among people with HIV who smoke cigarettes
Source: PLoS One. 2026 Aug 18;21(8):e0356296. doi: 10.1371/journal.pone.0356296 (PMC13485018; doi:10.1371/journal.pone.0356296)
Supplement: Supplementary file 4 [file pone.0356296.s004.docx]

**Supplementary Materials:**

**Trapezoidal Rule**

We estimated cumulative HIV viremia (Copies/year) for each participant up to the time of MI or censoring. This was done by applying the trapezoidal rule to all available viral load measurements prior to the event. The trapezoidal rule approximates the area under the curve by connecting consecutive viral load measurements with straight lines, effectively dividing the curve into a series of trapezoids. The area of each trapezoid is calculated and then summed to obtain the total cumulative viral load over time. This method allowed us to account for fluctuations in viral load across irregularly spaced time points, providing a more accurate estimate of the total HIV burden experienced by each participant.
